# Improvement in gross motor function and muscle tone in children with cerebral palsy related to neonatal icterus: an open-label, uncontrolled clinical trial

**DOI:** 10.1186/s12887-019-1669-2

**Published:** 2019-08-22

**Authors:** Liem Nguyen Thanh, Kien Nguyen Trung, Chinh Vu Duy, Doan Ngo Van, Phuong Nguyen Hoang, Anh Nguyen Thi Phuong, Minh Duy Ngo, Thinh Nguyen Thi, Anh Bui Viet

**Affiliations:** 1 Vinmec Research Institute of Stem Cell and Gene Technology, Hanoi, Vietnam; 2 Vinmec International Hospital, 458 Minh Khai Street, Hanoi, Vietnam

**Keywords:** Cerebral palsy, Stem cells, Neonatal icterus, Autologous bone marrow mononuclear cell transplantation

## Abstract

**Background:**

Although stem cell transplantation has been successfully performed for cerebral palsy (CP) related to oxygen deprivation, clinical trials involving the use of stem cell transplantation for CP related to neonatal icterus have not been reported. The aim of this study was to evaluate the effectiveness of transplantation of autologous bone marrow mononuclear cell (BMMC) for improving gross motor function and muscle tone in children with CP related to neonatal icterus.

**Methods:**

This open-label, uncontrolled clinical trial, which included 25 patients with CP related to neonatal icterus who had a Gross Motor Function Classification System (GMFCS) score between level II and level V, was conducted between July 2014 and July 2017 at Vinmec International Hospital (Vietnam). BMMC were harvested from the patients’ iliac crests. Two procedures involving BMMC transplantation via the intrathecal route were performed: the first transplantation was performed at baseline, and the second transplantation was performed 6 months after the first transplantation. Gross motor function and muscle tone were measured at three time points (baseline, 6 months, and 12 months) using the Gross Motor Function Measure (GMFM) and the Modified Ashworth Scale.

**Results:**

In this trial, we observed significant improvement in gross motor function and a significant decrease in muscle tone values. Total score on the 88-item GMFM (GMFM-88), scores on each GMFM-88 domain, and the 66-item GMFM (GMFM-66) percentile were significantly enhanced at 6 months and 12 months after the first transplantation compared with the corresponding baseline measurements (*p*-values < 0.05). In addition, a significant reduction was observed in muscle tone score after the transplantations (*p*-value < 0.05).

**Conclusion:**

Autologous BMMC transplantation can improve gross motor function and muscle tone in children with CP related to neonatal icterus.

**Trial registration:**

ClinicalTrials.gov identifier: NCT03123562. Retrospectively registered on December 26, 2017.

**Electronic supplementary material:**

The online version of this article (10.1186/s12887-019-1669-2) contains supplementary material, which is available to authorized users.

## Background

Neonatal icterus is a physiological condition that affects 60–70% of newborns worldwide [1]. It is estimated that 50% of term infants and 80% of preterm infants develop icterus, which typically manifests 2–4 days after birth. In general, neonatal icterus responds well to phototherapy, albumin infusion, or blood exchange [2, 3]. However, neonates with unconjugated hyperbilirubinemia can develop acute encephalopathy with focal necrosis of neurons and glia. Loss of axon neurites and myelin fibres and increased blood vessel density with poorly defined lumen structures have been observed in autopsied brain tissue from premature infants with kernicterus [4–12]. Acute bilirubin encephalopathy affects long-term neurodevelopmental outcomes. Bilirubin-induced damage to the brain can result in CP, deafness, and/or hearing loss [5–13]. In previous research, the risk for CP in neonates with hyperbilirubinemia was found to be 0.57 per 100,000 births [14].

The traditional treatment for CP is physiotherapy, which exhibits limited efficacy. The benefits of stem cell transplantation as a treatment for CP have recently been reported [15–36]. However, no clinical trials involving the use of stem cell transplantation for CP related to neonatal icterus have been reported. Since 2014, autologous BMMC transplantation for patients with CP related to neonatal icterus has been performed at Vinmec International Hospital. The aim of this study was to assess improvement in gross motor function and muscle tone in children with CP related to neonatal icterus after BMMC transplantation.

## Methods

### Patients

#### Inclusion criteria

- Sex: either sex.
- Age: from 2 to 15 years.
- Gross Motor Function Classification System [37] score: between level II and level V.
- Previous history of icterus during the neonatal period.

#### Exclusion criteria

- Coagulation disorder.
- Severe health condition such as cancer; failure of the heart, lungs, liver, or kidneys; or an active infection.

### Study design

An open-label, uncontrolled clinical trial.

### Research setting and duration

The study carried out at Vinmec Times City International Hospital from July 2014 to July 2017.

### Sample size

A study (2013) revealed that the mean of GMFM-66 score was 42.6 ± 15.59 [27]. We expected that it increases by 20.5% after 12 months intervention.

Alpha = 0.05, Power = 80%, *N* = 25.

During the study period, 33 patients with CP related to neonatal icterus were screened, 25 patients met the inclusion criterial.

### Clinical assessment

A comprehensive clinical examination with a particular focus on gross motor function and muscle tone was performed by an experienced physiotherapist at baseline and at 6 and 12 months after treatment. Gross motor function was classified into 5 different levels according to Gross Motor Function Classification System.

Changes in gross motor function were evaluated using the 88-item Gross Motor Function Measure (GMFM-88) [38], which consists of 88 items categorized into five domains: A. Lying and Rolling; B. Sitting; C. Crawling and Kneeling; D. Standing; E. Walking, Running and Jumping. The Gross Motor Ability Estimator (GMAE) was used to enter individual item scores, calculate GMFM-88 scores and convert these scores to 66-item Gross Motor Function Measure (GMFM-66) scores [39]. GMFM-66 percentile was used to illustrate the relative motor function of each participant compared to children of the same age with the same GMFCS level, excluding interference induced by improvement with age.

Muscle tone was assessed using the Modified Ashworth Scale [40].

The GMFM-88 and GMFM-66 percentiles were primary outcomes, and the Modified Ashworth Scale score was the secondary outcome.

### Laboratory and imaging diagnostics

All participants were tested for HIV, Hepatitis B virus, and Hepatitis C virus. Magnetic resonance imaging (MRI) and electroencephalography of the brain were also performed to assess the extent of brain injury.

### Isolation of BMMCs

Bone marrow aspiration was conducted under general anesthesia in an operating theater at Vinmec International Hospital. The required bone marrow volume was calculated in accordance with each participant’s body weight. Based on our prior experience, this volume was determined as follows: 8 mL/kg for patients who weighed less than 10 kg and [80 mL + (body weight in kg - 10) × 7 mL] for patients who weighed more than 10 kg, with a total volume of no more than 200 mL [16]. BMMC separation was performed using density gradient centrifugation with Ficoll [41]. The number of hematopoietic stem cells (CD34+ cells) and the viability of BMMC were evaluated using flow cytometry.

### Transplantation of BMMCs

Each patient underwent two BMMC transplantations, the first of which was performed immediately after harvested bone marrow was processed. The remaining BMMC were stored in liquid nitrogen at − 196 °C. The second transplantation was performed 6 months after the first transplantation. The average numbers of mononuclear cells and CD34+ cells per kg body weight were 17.4 ± 11.9 × 10^6^ and 1.5 ± 1.4 × 10^6^, respectively, for the first transplantation and 15.0 ± 12.8 × 10^6^ and 1.1 ± 1.1 × 10^6^, respectively, for the second transplantation. The average cell viabilities before the first and second transplantations were 96.9 and 71%, respectively. Each dose of cells was mixed with physiological saline to a volume of 10 mL for administration. Cells were then intrathecally infused into the space between the 4th and 5th lumbar vertebrae using an 18-gauge needle. This procedure was conducted in the recovery room by an experienced anesthesiologist and lasted 30 min.

### Rehabilitative therapy

After stem cell transplantation, all children received extensive rehabilitative therapy by rehabilitative physicians and physiotherapists for 12 days (1 h per day) at the rehabilitative center of Vinmec Times City International Hospital. Parents were instructed on how to perform continuous rehabilitative at home.

### Statistical analysis

Descriptive statistics are used to illustrate the demographics of children with CP related to neonatal icterus. Gross motor function and muscle tone at baseline, 6 months, and 12 months were compared using the Wilcoxon matched-pairs signed rank test.

A t-test was used to assess changes in the mean GMFM-88 score, GMFM-66 percentile, and Modified Ashworth Scale score at 12 months after stem cell transplantation by gender.

Changes in these mean scores by age group (< 36 months, 36–72 months, > 72 months) and GMFCS level were evaluated at 12 months after stem cell transplantation by one-way ANOVA. Bonferroni test in Post Hoc was used to determine the difference in the mean of each age group or the GMFCS level.

A *p*-value less than 0.05 was considered the threshold for significance. Data analyses were performed using STATA software version 12.0.

## Results

### Patients’ characteristics

Twenty-five patients with CP related to neonatal icterus, including 15 males and 10 females, were enrolled in this study. The median age for all study subjects was 5.4 years (range: 2–15 years). All patients suffered from bilateral spastic CP. The severities of patients’ conditions based on GMFCS level are presented in Table 1.

**Table 1:** Patient severity according to GMFCS classification

GMFCS level	Number	Percentage
II	2	8.0
III	3	12.0
IV	6	24.0
V	14	56.0

**Fig. 1:**
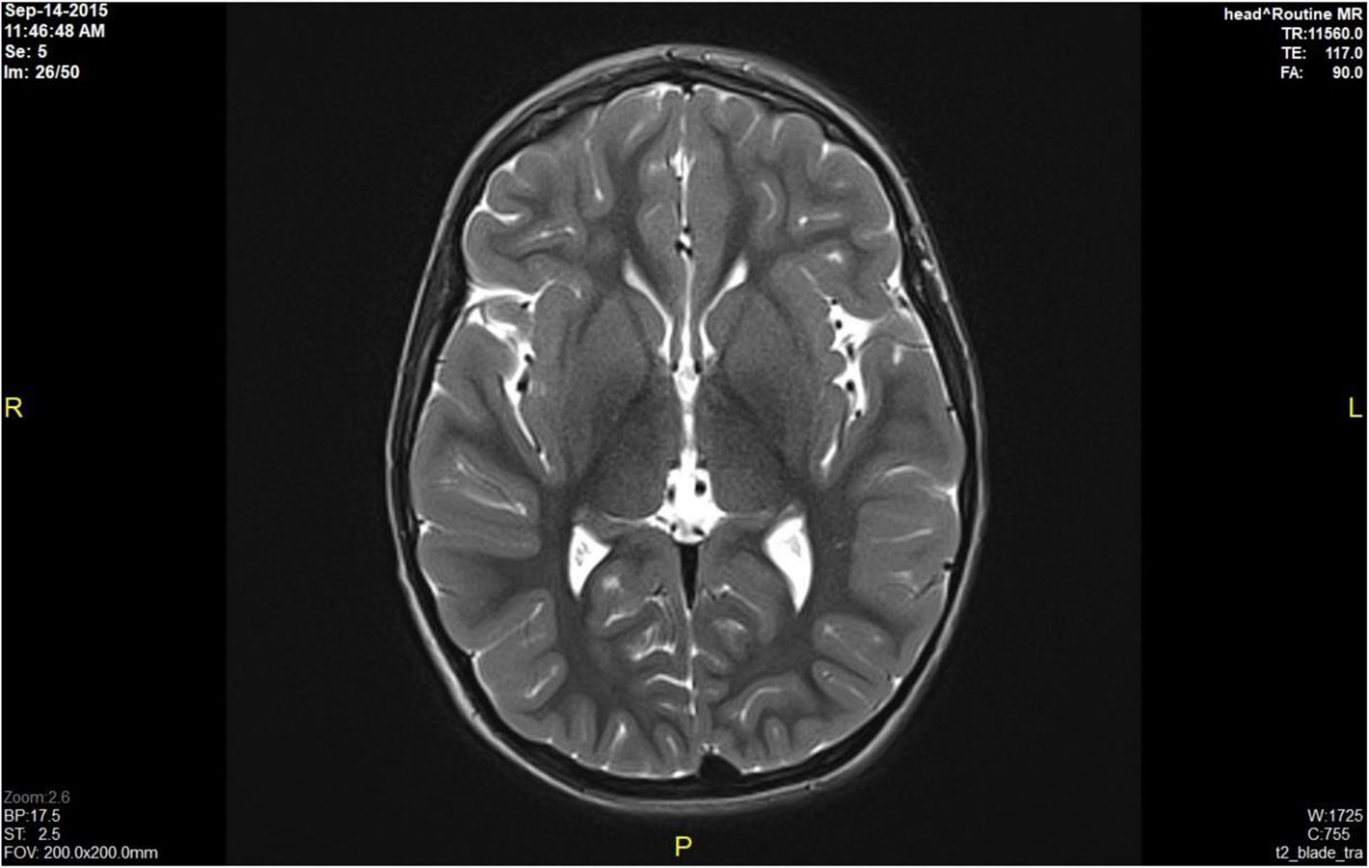
Normal brain

**Fig. 2:**
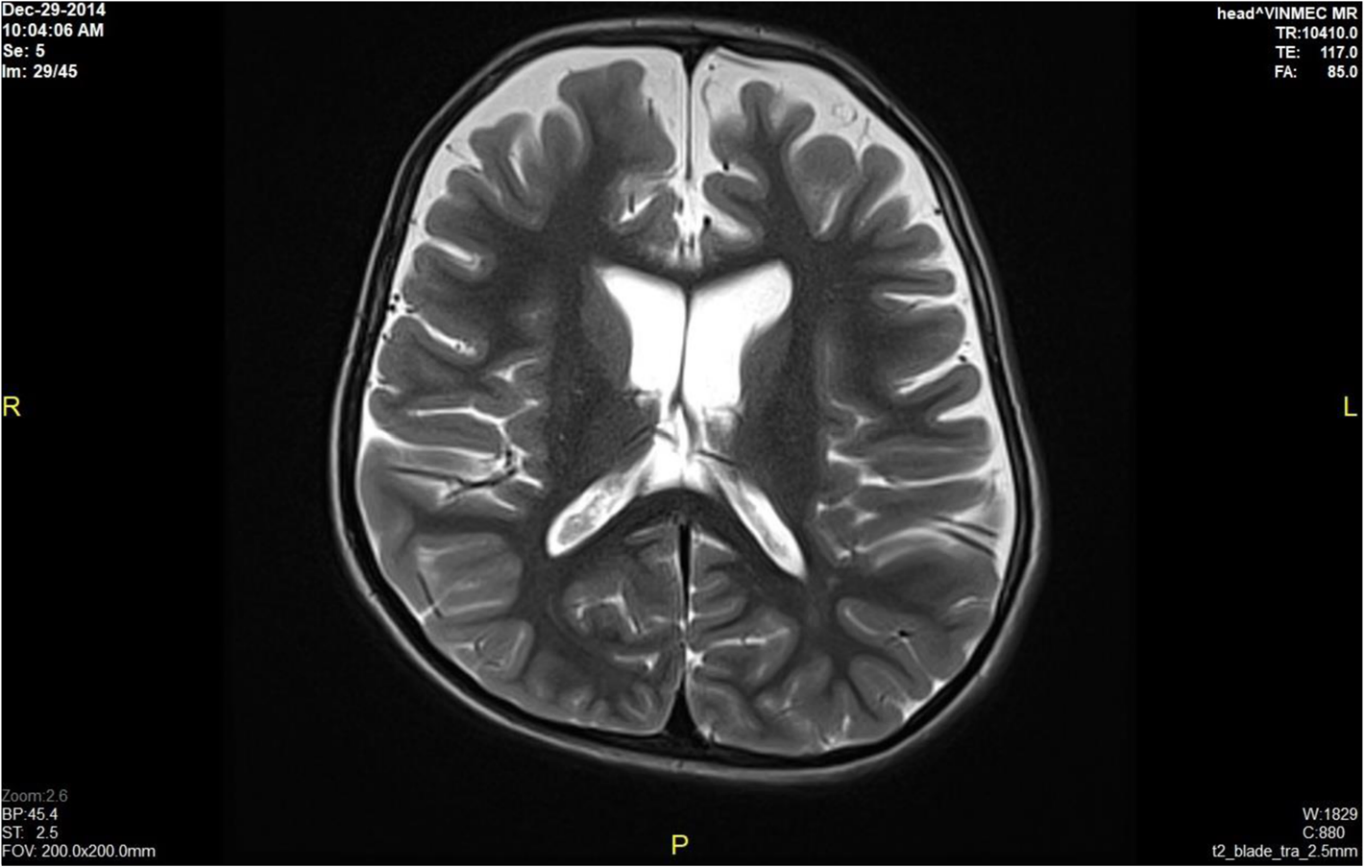
Mild cerebral atrophy in the supratentorial region

**Fig. 3:**
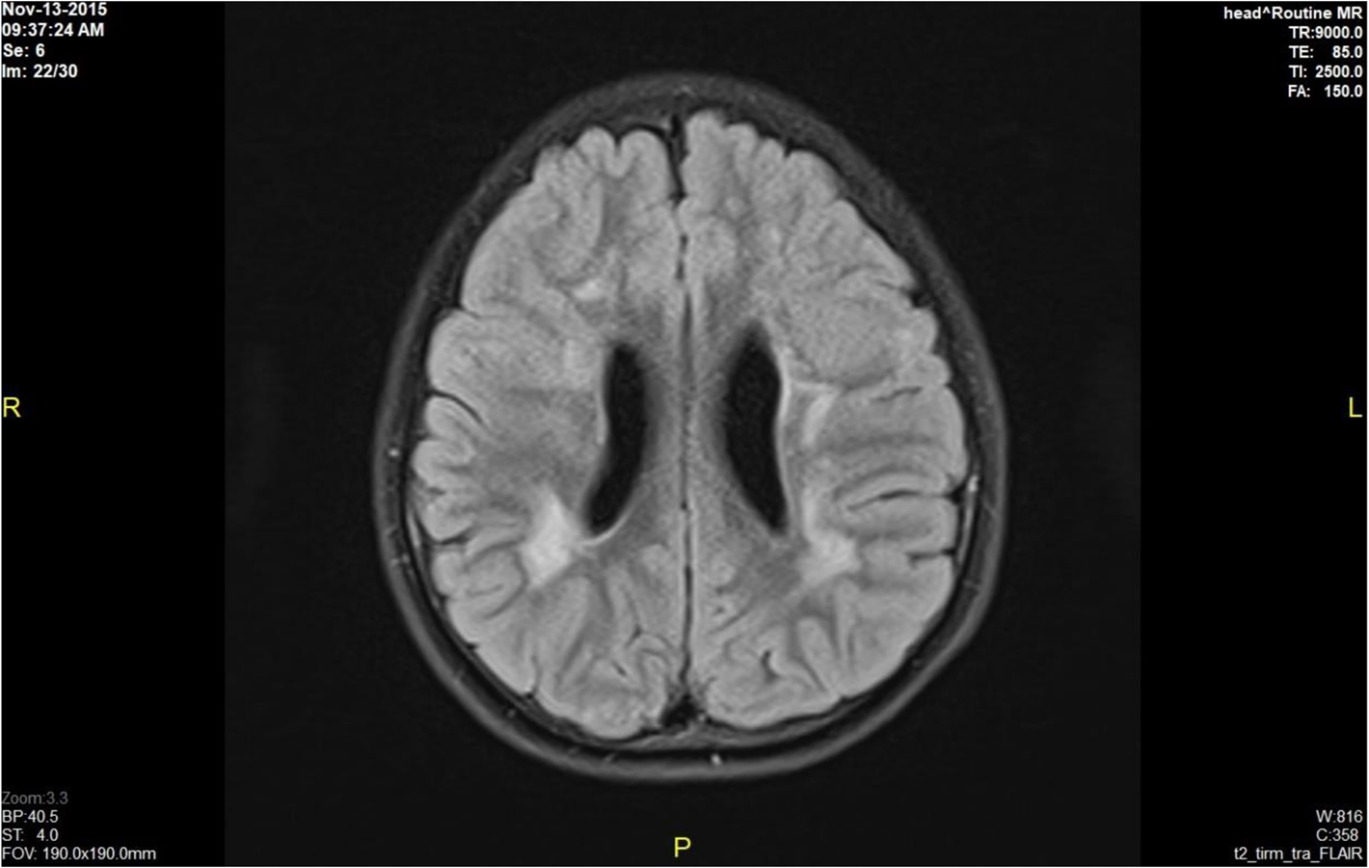
Periventricular white matter lesions

**Fig. 4:**
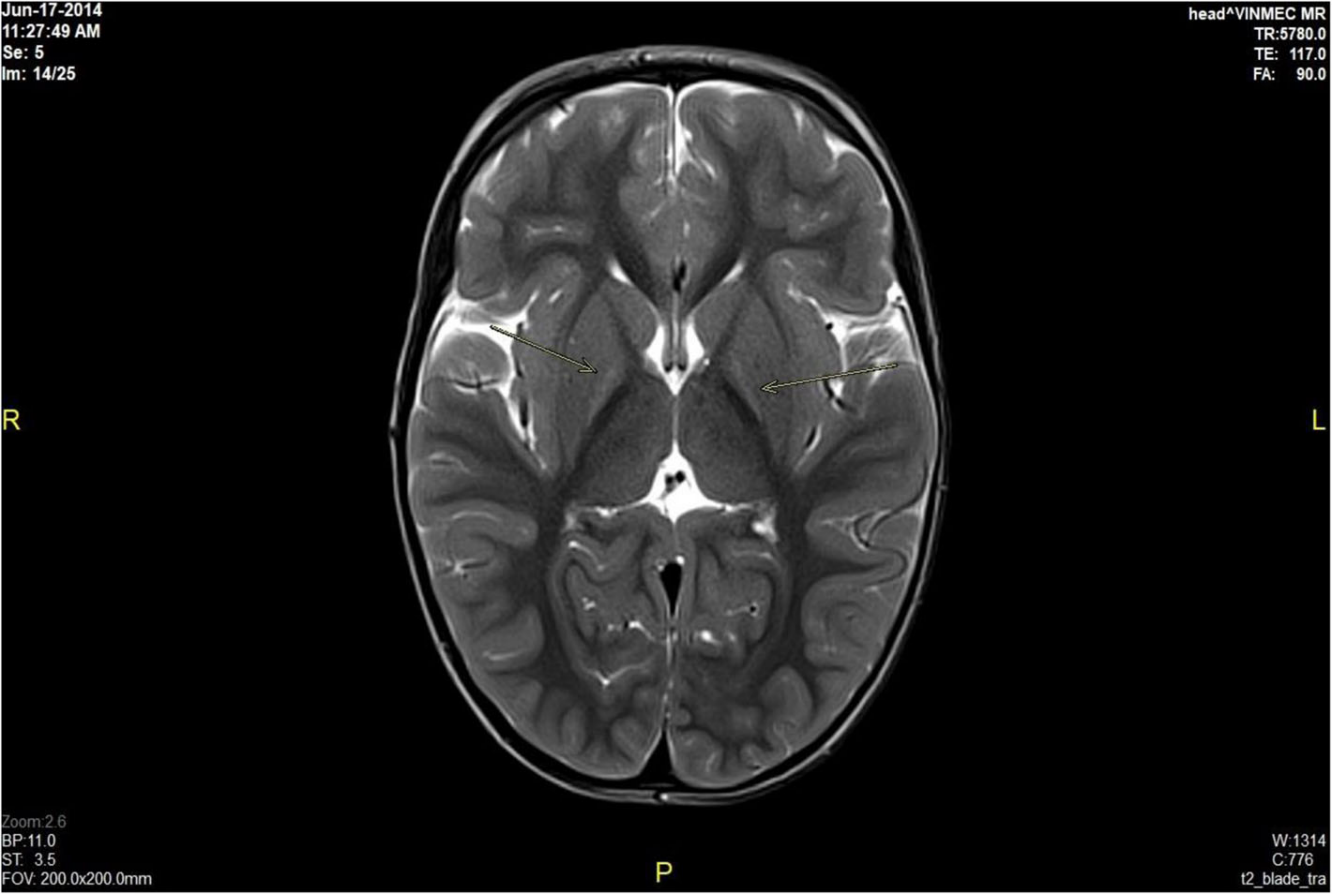
Bilateral globus pallidus lesions

Brain MRI results revealed bilateral globus pallidus lesions, mild cerebral atrophy in the supratentorial area, periventricular white matter lesions, and no abnormalities in 60, 8, 8, and 24% of the patients, respectively. Information related to MRI scans showing brain damage is described detail in Figs. 1, 2, 3 and 4 (Fig. 1 - Normal brain, Fig. 2 - Mild cerebral atrophy in the supratentorial region, Fig. 3 - Periventricular white matter lesions, Fig. 4 - Bilateral globus pallidus lesions).

### Gross motor function and muscle tone at baseline and at 6 and 12 months after stem cell transplantation

Overall, gross motor function was markedly improved at 6 and 12 months after stem cell transplantation, with median scores of 35.8 (27.6) and 53.2 (28.2), respectively, versus 18.3 (17.6) at baseline. The Wilcoxon matched-pairs signed rank test indicated that the GMFM-66, GMFM-88 and sub-domain median scores were significantly higher after transplantation than at baseline (*p*-value < 0.05).

The GMFM-66 percentile was significantly enhanced at 6 and 12 months after stem cell transplantation, with median scores of 22.5 (22.6) and 40.1 (5.5), respectively, compared to the median baseline score of 19.3 (19.6) (*p*-value < 0.05).

Muscle tone decreased significantly from a median Modified Ashworth Scale score of 4.0 (0.25) at baseline to 3.3 (0.63) at 6 months after stem cell transplantation and 3.0 (0.25) at 12 months after stem cell transplantation (*p*-values < 0.05). This observed improvement in gross motor functions and muscle tone is presented in greater detail in Table 2.

**Table 2:** Gross motor function improvement after stem cell transplantation

	Baseline Median (IQR)	6 months post-transplantation Median (IQR)	*p*-value at 6 months	12 months post-transplantation Median (IQR)	*p*-value at 12 months
Total GMFM-88 score	18.3 (17.6)	35.8 (27.6)	0.0003	53.2 (28.2)	0.0000
Lying and rolling	34.0 (26.0)	48.0 (10.0)	0.0011	51.0 (2.0)	0.0000
Sitting	15.0 (24.0)	38.0 (31.0)	0.0003	56.0 (9.0)	0.0000
Crawling and kneeling	0.0 (5.0)	9.0 (28.0)	0.0001	29.0 (27.0)	0.0000
Standing	0.0 (0.0)	0.0 (4.0)	0.0084	3.0 (14.0)	0.0003
Walking, running and jumping	0.0 (0.0)	0.0 (0.0)	0.0256	0.0 (5.0)	0.0084
GMFM-66 score	26.7 (14.8)	38.4 (12.9)	0.0003	45.6 (10.7)	0.0000
GMFM-66 percentile	19.3 (19.6)	22.5 (22.6)	0.0002	40.1 (5.5)	0.0000
Ashworth score	4.0 (0.25)	3.3 (0.63)	0.0000	3.0 (0.25)	0.0000

*IQR* Interquartile range

*Significant at *p* ≤ 0.05

### Relationships between patient characteristics and changes in gross motor function and muscle tone

The result showed no relationship between improvement in gross motor function and muscle tone based on patient age, sex, or GMFCS level (*p*-value > 0.05) (see details in Table 3).

**Table 3:** Changes in gross motor function and muscle tone after stem cell transplantation according to patient characteristics

Characteristics	Change in GMFM-88 score Mean [95% CI]		Change in GMFM-66 percentile Mean [95% CI]		Change in Ashworth score Mean [95% CI]	
Gender^a^						
Male	32.4 [25.2; 39.5]	*p*-value = 0.808	20.2 [16.7; 23.6]	*p*-value = 0.593	1.0 [0.7; 1.2]	*p*-value = 0.301
Female	31.1 [26.8;36.9]		18.7 [13.5; 23.9]		0.8 [0.3; 1.2]	
Age^b^						
< 36 months	37.1 [31.9; 42.3]	*p*-value = 0.368	21.7 [16.2; 27.1]	*p*-value = 0.776	0	*p*-value = 0.393
36–72 months	30.9 [22.4; 39.5]		17.9 [13.7; 22.1]		0.8 [0.6; 1.0]	
> 72 months	31.9 [25.1; 38.7]		21.0 [17.1; 25.0]		0.9 [0.5; 1.4]	
GMFCS^b^						
Level II	8.9 [6.0; 11.8]	*p*-value = 0.574	9.6 [1.1; 18.1]	*p*-value = 0.929	0.8 [0.7; 0.9]	*p*-value = 0.316
Level III	21.1 [12.6; 29.5]		13.9 [6.9; 20.8]		0.6 [0.04; 1.2]	
Level IV	39.5 [31.8; 47.2]		18.6 [15.0; 22.2]		1.0 [0.9; 1.0]	
Level V	34.2 [28.9; 39.4]		22.6 [19.5; 25.7]		0.9 [0.6; 1.3]	

^a^t-test

^b^one-way ANOVA test (Bonferroni test for Post Hoc analysis)

### Adverse events

No severe complications occurred during the study period. Minor complications occurred and were managed with standard medications. Adverse events included vomiting (32%), local pain (16%), and mild fever without any identified infection (4%).

## Discussion

To our knowledge, this report describes the first clinical trial to assess the impact of autologous BMMC transplantation on motor function and muscle tone in children with CP related to neonatal icterus.

Overall, observations from this clinical trial indicate that gross motor function was significantly improved at 6 and 12 months after stem cell transplantation. The GMFM-88 score increased by 17.5 and 34.9 at 6 months, and 12 months after transplantation than that at baseline, respectively. This level of improvement was higher than the study of Wang [27] but lower than our previous study [16]. The GMFM-88 score in Wang’s study increased by 7.89 at 6 months after transplantation than baseline scores. The GMFM-88 in our study in using stem cell transplantation for CP related to oxygen deprivation increased by 25.1 at 6 months after the transplantation.

In 2016, Kulak conducted a systematic review 7 studies on stem cell treatment for cerebral palsy with fives studies using the GMFM-88 score as a primary outcome. The improvement in the GMFM-88 scale was noted in all five studies after transplantation. However, the causes of cerebral palsy were not identified in those study [42].

In accordance to previous studies using stem cell transplantation for children with CP, we observed a significant reduction in the median Ashworth score in patients at 6 and 12 months after transplantation [16, 20, 22], indicating the effectiveness of the therapy on muscle spastic reduction in the patients.

Our results indicated that autologous intrathecal BMMC transplantation was safe for children with CP related to neonatal icterus. No complications occurred during bone marrow harvesting or BMMC infusion. During hospitalization after stem cell transplantation, only 4.2% of the patients exhibited mild fever with no signs of infection, and 34% of the patients experienced intermittent vomiting. All adverse events were easily managed with medical treatment. These findings were similar to those obtained in previous clinical trials in which autologous BMMC transplantation was used to treat for children with CP [16, 20, 22].

Adverse events were less severe in our series than in previously reported trials that involved the use of allogenic stem cells from umbilical cord blood. In such studies, severe adverse events such as pneumonia, influenza, urinary tract infection and even death were observed [32]. One explanation for this difference could be suppression of the immune system due to the use of immunosuppressive medications in allogenic umbilical cord blood transplantations. In our patients, stem cells were administered via the intrathecal route, as described in our previous report [16]. The outcomes again confirmed that this route is minimally invasive, safe and effective.

This study has some limitations. There was no control group. In addition, the follow-up time of 6 months after the 2nd stem cell transplantation was relatively short.

## Conclusions

Based on the results of this study, we can conclude that gross motor function and muscle tone in children with CP related to neonatal icterus were remarkably improved at 6 months and 12 months after BMMC transplantation. However, these finding should be confirmed in larger, multicenter, placebo-controlled trials.

## Additional file

Additional file 1: Stem cell transplantation for children with cerebral palsy related to neonatal icterus. This is a dataset file of study on autologous bone marrow mononuclear cells for cerebral palsy related to neonatal icterus. The data includes demographic data, gross motor function of patients with cerebral palsy related to neonatal icterus such as GMFM-88 total score, GMFM-66 percentile. The dataset consists of 25 observations and 93 variables. (XLSX 20 kb)

## Data Availability

All data generated or analyzed during this study are included in this published article and its Additional file 1.

## Abbreviations

BMMC: Bone marrow mononuclear cell
CP: Cerebral palsy
GMAE: Gross Motor Ability Estimator
GMFCS: Gross Motor Function Classification System
GMFM: Gross Motor Function Measure
MRI: Magnetic resonance imaging
